# A space–time spectral collocation algorithm for the variable order fractional wave equation

**DOI:** 10.1186/s40064-016-2899-5

**Published:** 2016-08-02

**Authors:** A. H. Bhrawy, E. H. Doha, J. F. Alzaidy, M. A. Abdelkawy

**Affiliations:** 1 Department of Mathematics, Faculty of Science, Beni-Suef University, Beni-Suef, Egypt; 2 Department of Mathematics, Faculty of Science, Cairo University, Giza, 12613 Egypt; 3 Department of Mathematics, Faculty of Science, King Abdulaziz University, Jeddah, Saudi Arabia; 4 Department of Mathematics and Statistics, College of Science, Al-Imam Mohammad Ibn Saud Islamic University (IMSIU), Riyadh, Saudi Arabia

**Keywords:** Variable-order fractional derivative, Collocation method, Jacobi polynomials, Gauss quadrature, Fractional wave equation

## Abstract

The variable order wave equation plays a major role in acoustics, electromagnetics, and fluid dynamics. In this paper, we consider the space–time variable order fractional wave equation with variable coefficients. We propose an effective numerical method for solving the aforementioned problem in a bounded domain. The shifted Jacobi polynomials are used as basis functions, and the variable-order fractional derivative is described in the Caputo sense. The proposed method is a combination of shifted Jacobi–Gauss–Lobatto collocation scheme for the spatial discretization and the shifted Jacobi–Gauss–Radau collocation scheme for temporal discretization. The aforementioned problem is then reduced to a problem consists of a system of easily solvable algebraic equations. Finally, numerical examples are presented to show the effectiveness of the proposed numerical method.

## Background

The subject of fractional calculus is one of the branches of applied mathematics which deals with derivatives and integrals of any arbitrary order (Hilfer 2000; Kilbas and Trujillo 2002; Kilbas et al. 2006). Fractional partial differential equations are describing the phenomena in many various areas such as fluid mechanics, physics, engineering, biology (Miller and Ross 1993; Giona and Roman 1992; Rossikhin and Shitikova 1997; Podlubny 1999; West 2007). The concept of variable-order fractional allows the power of the fractional operator to be a function of the independent variable (Coimbra 2003; Chechkin et al. 2005; Evans and Jacob 2007; Sun et al. 2009; Coimbra et al. 2005; Coimbra and Ramirez 2007). Few numerical methods have been introduced and discussed to solve the variable-order fractional problems (Sun et al. 2012; Ma et al. 2012; Zeng et al. 2015; Fu et al. 2015; Abdelkawy et al. 2015). Bhrawy and Zaky (2015a) proposed a new algorithm for solving one-and two-dimensional variable-order cable equations based on Jacobi spectral collocation approximation together with the Jacobi operational matrix for variable-order fractional derivative. Chen et al. (2014) proposed an implicit alternating direct method for the two-dimensional variable-order fractional percolation equation also discussed the stability and convergence of the implicit alternating direct method.

Spectral methods (Canuto et al. 2006; Saadatmandi and Dehghan 2011; Doha and Bhrawy 2012; Bhrawy and Zaky 2015b, c; Bhrawy et al. 2016a) have been widely used in many fields in the last four decades. In the early times, the spectral method based on Fourier expansion has been used in few fields such as a simple geometric field and periodic boundary conditions. Recently, they have been developed theoretically and used as powerful techniques to solve various kinds of problems. Based on the accuracy and exponential rates of convergence, spectral methods have an excellent reputation when compared with others numerical methods. The expression of the problem solution as a finite series of polynomials/functions is the major step of all types of spectral methods. Then, the coefficients of this expansion will be chosen such that the absolute error is diminished as well as possible.

The spectral collocation method (Canuto et al. 2006; Bhrawy and Alofi 2013; Gu and Chen 2014; Bhrawy and Abdelkawy 2015; Bhrawy 2016a) is a specific type of spectral methods, that is more applicable and widely used to solve almost types of differential (Bhrawy et al. 2016b; Tatari and Haghighi 2014), integral (Bhrawy et al. 2016c; Rahmoune 2013), integro-differential (Jiang and Ma 2013; Ma and Huang 2014) and delay differential (Bhrawy et al. 2015a; Reutskiy 2015) equations. While, the numerical solution will be enforced to almost satisfy the partial differential equations (PDEs) in spectral collocation method. In other words, the residuals may be permitting to be zero at chosen points. Wei and Chen (2012) proposed Legendre spectral collocation methods for pantograph Volterra delay-integro-differential equations. Bhrawy and Alofi (2012) introduced the spectral shifted Jacobi–Gauss collocation method for solving the Lane–Emden type equation. Bhrawy et al. (2015b) proposed the spectral collocation algorithm to solve numerically some wave equations subject to initial-boundary nonlocal conservation conditions in one and two space dimensions. Bhrawy (2016b) proposed Jacobi spectral collocation method for solving multi-dimensional nonlinear fractional sub-diffusion equations.

The aim of this paper is to find the numerical solution of the space–time variable order fractional wave equation subject to initial-boundary conditions. The wave equation is an important second-order partial differential equation for the description of waves as they occur in physics such as sound waves, light waves and water waves. Variable order wave equation appears in areas such as acoustics, electromagnetics, and fluid dynamics. This paper extends the SJ–GL-C and SJ–GR-C schemes in order to solve the space-time variable order fractional wave equation. The proposed collocation scheme is investigated for both temporal and spatial discretizations. The SJ–GL-C and SJ–GR-C are proposed, with a suitable modification for treating the boundary and initial conditions, for spatial and temporal discretizations. This treatment, for the conditions, improves the accuracy of the scheme greatly. Therefore, the space–time variable order fractional wave equation with its conditions is reduced to system of algebraic equations which is far easier to be solved. Finally, numerical examples with comparisons lighting the high accuracy and effectiveness of the proposed algorithm are presented.

The present paper is presented as follows. The definitions of the fractional calculus and some properties of Jacobi polynomials are introduced in “Preliminaries” section. The spectral collocation methods for the space–time variable order fractional wave problem subject to initial-boundary conditions are presented in “Jacobi collocation method” section and then illustrated with two examples in “Numerical examples” section. The “Conclusion” is included in the last section.

## Preliminaries

We first recall some definitions and preliminaries of the variable-order fractional differential and integral operators and some knowledge of orthogonal shifted Jacobi polynomials that are most relevant to spectral approximations.

### **Definition 1**

The Riemann–Liouville and Caputo differential operators of constant order $\gamma ,$ when $n-1\le \gamma <n,$ of *f*(*t*) are given respectively by,

1 $$\begin{aligned} \begin{aligned}&{}_0 D_t^\gamma f(t)= \frac{1}{{\Gamma (n - \gamma )}}\frac{{d^n }}{{dt^n }}\int \limits _0^t {\frac{{f(s)}}{{(t - s )^{\gamma - n + 1} }}} ds,\\&{}_0^C D^{\gamma }_tf(t) = \frac{1}{{\Gamma (n - \gamma )}}\int \limits _0^t {\frac{{f^{(n)} (s )}}{{(t - s )^{\gamma - n + 1} }}} ds, \end{aligned} \end{aligned}$$

where $\Gamma (.)$ represents the Euler gamma function.

### **Definition 2**

The left Riemann–Liouville variable-order fractional differential operator of order $\gamma (t)$ is given by

2 $$\begin{aligned} {}_0 D_t^{\gamma (t)} f(t) = \frac{1}{{\Gamma (n - \gamma (t))}}\frac{{d^n }}{{dt^n }}\int \limits _0^t {\frac{{f(s)}}{{(t - s)^{\gamma (t) - n + 1} }}ds,} \end{aligned}$$

where $n-1< \gamma _{\min }< \gamma (t)< \gamma _{\max } < n , n \in \mathbb {N}$ for all $t \in [0,\tau ]$.

### **Definition 3**

The Caputo variable-order fractional differential operator is given by

3 $$\begin{aligned} {}_0^{C} D_t^{\gamma (t)} f(t) = \frac{1}{{\Gamma (1 - \gamma (t))}}\int \limits _{0 }^t {\frac{{f'(s)}}{{(t - s)^{\gamma (t)} }}ds }, \end{aligned}$$

where $0< \gamma (t) \le 1$ for all $t \in [0,\tau ]$.

It is important to note here that the constant-order fractional derivative can be seen as a special case of the variable-order fractional derivative. These two definitions are related by the following relation:

4 $$\begin{aligned} {}_0 D_t^{\gamma (t)}f(t) = \sum \limits _{k = 0}^{n - 1} {\frac{{f^{(k)} (0)t^{k - \gamma (t)} }}{{\Gamma (k + 1 - \gamma (t))}}} + \ {}_0^{C} D_t^{\gamma (t)} f(t). \end{aligned}$$

The Jacobi polynomials, denoted by $P_{j}^{(\theta ,\vartheta )}(x) (j=0,1\ldots ); \theta>-1, \vartheta >-1$ and defined on the interval $[-1,1]$ are generated from the three-term recurrence relation:

$$\begin{aligned} \begin{aligned}&P^{(\theta ,\vartheta )}_{i+1}(x)=\left( a^{(\theta ,\vartheta )}_i x-b^{(\theta ,\vartheta )}_i\right) P^{(\theta ,\vartheta )}_{i}(x)-c^{(\theta ,\vartheta )}_i P^{(\theta ,\vartheta )}_{i-1}(x),\quad \quad i\ge 1,\\&P^{(\theta ,\vartheta )}_{0}(x)=1,\quad P^{(\theta ,\vartheta )}_{1}(x)=\frac{1}{2 }(\theta +\vartheta +2)x+\frac{1}{2}(\theta -\vartheta ), \end{aligned} \end{aligned}$$

where

$$\begin{aligned} \begin{aligned} a^{(\theta ,\vartheta )}_i &=\frac{(2i+\theta +\vartheta +1)(2i+\theta +\vartheta +2)}{2(i+1)(i+\theta +\vartheta +1)},\\ b^{(\theta ,\vartheta )}_i &= \frac{(2i+\theta +\vartheta +1)({\vartheta }^2-{\theta }^2)}{2(i+1)(i+\theta +\vartheta +1)(2i+\theta +\vartheta )},\\ c^{(\theta ,\vartheta )}_i &= \frac{(2i+\theta +\vartheta +2)(i+\theta )(i+\vartheta )}{(i+1)(i+\theta +\vartheta +1)(2i+\theta +\vartheta )}. \end{aligned} \end{aligned}$$

The formula that relates Jacobi polynomials and their derivatives is

5 $$\begin{aligned} \begin{aligned} D^{(q)} P_{k}^{(\theta ,\vartheta )}(x)= P_{k}^{(\theta ,\vartheta ,q)}(x)=2^{-q}\frac{\Gamma (k+\theta +\vartheta +q+1)}{\Gamma (k+\theta +\vartheta +1)}P_{k-q}^{(\theta +q,\vartheta +q)}(x). \end{aligned} \end{aligned}$$

The orthogonality condition is

6 $$\begin{aligned} (P_{k}^{(\theta ,\vartheta )}(x),P_{l}^{(\theta ,\vartheta )}(x))_{w^{(\theta ,\vartheta )} }=\int \limits _{-1}^{1}P_{k}^{(\theta ,\vartheta )}(x) P_{l}^{(\theta ,\vartheta )}(x) \, w^{(\theta ,\vartheta )} (x)dx =h_k^{(\theta ,\vartheta )}\delta _{lk}, \end{aligned}$$

where $$w^{(\theta ,\vartheta )}=(1-x)^\theta (1+x)^\vartheta , h_{k}^{(\theta ,\vartheta )} =\dfrac{2^{\theta +\vartheta +1}\Gamma (k+\theta +1)\Gamma (k+\vartheta +1)}{(2k+\theta +\vartheta +1) k!\Gamma (k+\theta +\vartheta +1)}.$$

Let the shifted Jacobi polynomials $P^{(\theta ,\vartheta )}_i{(\dfrac{2x}{L}-1)}$ be denoted by $P^{(\theta ,\vartheta )}_{L,i}{(x)}$, then they can be obtained with the aid of the following recurrence formula:

7 $$\begin{aligned} \begin{aligned}&P_{L,i+1}^{(\theta ,\vartheta )}{(x)}=\left( a_{i}^{(\theta ,\vartheta )} \left( \frac{2x}{L}-1\right) -b_{i}^{(\theta ,\vartheta )}\right) P_{L,i}^{(\theta ,\vartheta }{(x)}-c_{i}^{(\theta ,\vartheta )} P_{L,i-1}^{(\theta ,\vartheta )}{(x)},\quad i\ge 1,\\&P_{L,0}^{(\theta ,\vartheta )}{(x)}=1,\quad P_{L,1}^{(\theta ,\vartheta )}{(x)}=\frac{1}{L}(\theta +\vartheta +2)x-(\vartheta +1), \end{aligned} \end{aligned}$$

The analytic form of the shifted Jacobi polynomials $P^{(\theta ,\vartheta )}_{L,i}{(x)}$ of degree *i* is given by

8 $$\begin{aligned} P_{L,i}^{(\theta ,\vartheta )}{(x)}=\sum \limits _{k=0}^{i }{(-1)}^{i+k}\frac{ \Gamma {(i+\vartheta +1)}\Gamma {(i+k+\theta +\vartheta +1)}}{\Gamma {(k+\vartheta +1)}\Gamma {(i+\theta +\vartheta +1)}(i-k)!k!\, L^k}\ x^k, \end{aligned}$$

and the orthogonality condition is

9 $$\begin{aligned} \int _{0}^{L} P^{(\theta ,\vartheta )}_{L,j} (x)P^{(\theta ,\vartheta )}_{L,k}(x) w^{(\theta ,\vartheta )}_{L} (x)dx =\hbar ^{(\theta ,\vartheta )}_{L,k}\, \delta _{jk}, \end{aligned}$$

where $$w_{L}^{(\theta ,\vartheta )} (x)={x}^{\vartheta }(L-x)^{\theta }$$ and $$\hbar ^{(\theta ,\vartheta )}_{L,k} =\dfrac{L^{\theta +\vartheta +1}\Gamma (k+\theta +1)\Gamma (k+\vartheta +1)}{(2k+\theta +\vartheta +1) k!\Gamma (k+\theta +\vartheta +1)}$$.

The shifted Jacobi–Gauss quadrature is commonly used to evaluate the previous integrals accurately. For any $\phi \in S_{2N+1}[0,L]$, we have

$$\begin{aligned} \int _0^L {\phi (x)w_{L}^{(\theta ,\vartheta )} (x)dx} = \sum \limits _{j = 0}^N {\varpi _{G,L,j}^{(\theta ,\vartheta )} \phi \left( x_{G,L,j}^{(\theta ,\vartheta )} \right) }, \end{aligned}$$

where $S_{N}[0,L]$ is the set of polynomials of degree less than or equal to $N, x_{G ,L,j}^{(\theta ,\vartheta )}\ (0\le j \le N )$ and $\varpi _{G ,L,j}^{(\theta ,\vartheta )}\ (0\le j \le N )$ are used as usual the nodes and the corresponding Christoffel numbers in the interval [0, *L*], respectively.

For shifted Jacobi–Gauss (SJ–G), $x_{G ,L,j}^{(\theta ,\vartheta )}\ (0\le j \le N )$ are the zeros of $P_{L,N+1}^{(\theta ,\vartheta )}(x)$ and the weights

10 $$\begin{aligned} \begin{aligned} \varpi _{G ,L,j}^{(\theta ,\vartheta )}=\frac{{C_{L,N}^{(\theta ,\vartheta )} }}{{\left( L - x_{G ,L,j}^{(\theta ,\vartheta )}\right) \,x_{G ,L,j}^{(\theta ,\vartheta )} \left[ {\partial _x P_{N + 1}^{(\theta ,\vartheta )} \left( x_{G ,L,j}^{(\theta ,\vartheta )}\right) } \right] ^2 }},\quad 0 \le j \le N, \end{aligned} \end{aligned}$$

where

$$\begin{aligned} C_{L,N}^{(\theta ,\vartheta )} = \frac{{L^{\theta + \vartheta + 1} \Gamma (N + \theta + 2)\Gamma (N + \vartheta + 2)}}{{(N + 1)!\Gamma (N + \theta + \vartheta + 2)}}. \end{aligned}$$

while the nodes and the corresponding Christoffel numbers in the shifted Jacobi Gauss–Radau (SJ–GR) quadrature are given by $x_{R ,L,0}^{(\theta ,\vartheta )}=0,\; x_{R ,L,j}^{(\theta ,\vartheta )}\ (1\le j \le N )$ are the zeros of $P_{L,N}^{(\theta ,\vartheta +1)}(x),$ and the weights

11 $$\begin{aligned} \begin{aligned} \varpi _{R ,L,0}^{(\theta ,\vartheta )}&=\frac{{(L )^{\theta + \vartheta + 1} (\vartheta + 1) \Gamma ^2 (\vartheta + 1)\Gamma (N+1)\Gamma (N + \theta + 1)}}{{\Gamma (N + \vartheta + 2)\Gamma (N + \theta + \vartheta + 2)}},\\ \varpi _{R ,L,j}^{(\theta ,\vartheta )}&=\frac{{C_{L,N - 1}^{(\theta ,\vartheta + 1)} }}{{\left( L - x_{R ,L,j}^{(\theta ,\vartheta )}\right) \left( x_{R ,L,j}^{(\theta ,\vartheta )}\right) ^2 \partial _x \left[ {P_N^{(\theta ,\vartheta + 1)} \left( x_{R ,L,j}^{(\theta ,\vartheta )}\right) } \right] ^2 }},\quad 1 \le j \le N, \end{aligned} \end{aligned}$$

A function *u*(*x*), square integrable in [0, *L*], may be expressed in terms of shifted Jacobi polynomials as

$$\begin{aligned} u{(x)}=\sum \limits _{j=0}^{\infty }c_j P^{(\theta ,\vartheta )}_{L,j}(x), \end{aligned}$$

where the coefficients $c_j$ are given by

12 $$\begin{aligned} c_j= \dfrac{1}{ \hbar ^{(\theta ,\vartheta )}_{L,j}}\int _{0}^{L}u (x)P^{(\theta ,\vartheta )}_{L,j}(x)w_{L}^{(\theta ,\vartheta )}(x)dx, \quad j=0,1,2, \ldots . \end{aligned}$$

The *q*th derivative of $P_{L,k}^{(\theta ,\vartheta )}(x)$ can be written as

13 $$\begin{aligned} D^{q}P_{L,k}^{(\theta ,\vartheta )}{(x)}=P_{L,k}^{(\theta ,\vartheta ,q)}{(x)}=\frac{ \Gamma (q+k+\theta +\vartheta +1)}{L^{q}\Gamma (k+\theta +\vartheta +1)}P_{L,k-q}^{(\theta +q,\vartheta +q)}{(x)}. \end{aligned}$$

Accordingly, we can calculate the Caputo variable order derivative of shifted Jacobi polynomials from

14 $$\begin{aligned} \begin{aligned} {}^{C} D_x^{\gamma (x)} P_{L,i}^{(\theta ,\vartheta )}{(x)}&=P_{L,i}^{(\theta ,\vartheta ,\gamma (x))}{(x)}\\&=\sum \limits _{k=1}^{i }\frac{ {(-1)}^{i+k}\Gamma {(i+\vartheta +1)}\Gamma {(i+k+\theta +\vartheta +1)}}{\Gamma {(k+\vartheta +1)}\Gamma {(i+\theta +\vartheta +1)}(i-k)!\, L^k\ \Gamma {(k-\gamma (x)+1)}}\ x^{k-\gamma (x)}. \end{aligned} \end{aligned}$$

## Jacobi collocation method

In this section, we introduce a numerical algorithm extends the SJ–GL-C and SJ–GR-C schemes in order to solve the space-time variable order fractional wave equation. The collocation points are selected at the SJ–GR and SJ–GL interpolation nodes for temporal and spatial variables, respectively. The core of the proposed method consists of discretizing the space–time variable order fractional wave equation to create a system of algebraic equations of the unknown coefficients. This system can be then easily solved with a standard numerical scheme.

In particular, we consider the following space–time variable order fractional wave equation

15 $$\begin{aligned} \begin{aligned} {}^{C} D_{t}^{\beta (x,t)}u(x,t)=B(x,t){}^{C} D_{x}^{\alpha (x,t)}u(x,t)+f(u,x,t),\quad 1<\alpha (x,t),\beta (x,t)\le 2, \end{aligned} \end{aligned}$$

with the initial conditions

16 $$\begin{aligned} \begin{aligned} u(x,0)=g_{0}(x),\quad u_{t}(x,0)=g_{1}(x), \quad x\in [0,L], \end{aligned} \end{aligned}$$

and the boundary conditions

17 $$\begin{aligned} \begin{aligned} u(0,t)=g_{2}(t),\quad u(L,t)=g_{3}(t), \quad t\in [0,T] \end{aligned} \end{aligned}$$

where $B(x,t)>0, g_{0}(x),g_{1}(x),g_{2}(t)$ and $g_{3}(t)$ are given functions, while *f*(*u*, *x*, *t*) is a source term.

We choose the approximate solution to be of the form

18 $$\begin{aligned} \begin{aligned} u(x,t)&=\sum \limits _{i,j=0}^N{\hat{u}_{i,j}\, P_{L,i}^{(\theta {_{1}},\vartheta _{1})}(x)\, P_{T,j}^{(\theta _{2},\vartheta _{2})}(t)},\\&=\sum \limits _{i,j=0}^N \hat{u}_{i,j}\,\mathcal {P}_{0}^{i,j}(x,t), \end{aligned} \end{aligned}$$

where $$\mathcal {P}_{0}^{i,j,k}(x,y,t)=P_{L,i}^{(\theta _{1},\vartheta _{1})}(x)\, P_{T,j}^{(\theta _{2},\vartheta _{2})}(t).$$

The approximation of the temporal partial derivative $D_{t}u(x,t)$ can be easily computed as follows

19 $$\begin{aligned} \begin{aligned} D_{t}u(x,t)&=\sum \limits _{i,j = 0}^N\hat{u}_{i,j}\, P_{L,i}^{(\theta _{1},\vartheta _{1})}(x)\, P_{T,j}^{(\theta _{2},\vartheta _{2},1)}(t)\\&=\sum \limits _{i,j = 0}^N\hat{u}_{i,j}\,\mathcal {P}_{1}^{i,j}(x,t), \end{aligned} \end{aligned}$$

where $$\mathcal {P}_{1}^{i,j}(x,t)=P_{L,i}^{(\theta _{1},\vartheta _{1})}(x)\,P_{T,j}^{(\theta _{2},\vartheta _{2},1)}(t).$$

A straightforward calculation shows that the fractional derivative of variable order of the approximate solution can be computed by

20 $$\begin{aligned} {}^{C} D_{t}^{\beta (x,t)} u(x,t) & = {} \sum \limits _{i,j = 0}^N\hat{u}_{i,j}\, P_{L,i}^{(\theta _{1},\vartheta _{1})}(x)\, P_{T,j}^{(\theta _{2},\vartheta _{2},{\beta (x,t)})}(t)\nonumber \\& = {} \sum \limits _{i,j = 0}^N\hat{u}_{i,j}\,\mathcal {P}_{2}^{i,j}(x,t),\end{aligned}$$

21 $$\begin{aligned} {}^{C} D_{x}^{\alpha (x,t)} u(x,t)& = {} \sum \limits _{i,j= 0}^N\hat{u}_{i,j}\, P_{L,i}^{(\theta _{1},\vartheta _{1},{\alpha (x,t)})}(x)\, P_{T,j}^{(\theta _{2},\vartheta _{2})}(t)\nonumber \\& = {} \sum \limits _{i,j = 0}^N\hat{u}_{i,j}\,\mathcal {P}_{3}^{i,j}(x,t), \end{aligned}$$

where

$$\begin{aligned} \mathcal {P}_{2}^{i,j}(x,t)& = {} P_{L,i}^{(\theta _{1},\vartheta _{1})}(x)\, P_{T,j}^{(\theta _{2},\vartheta _{2},{\beta (x,t)})}(t),\\ \mathcal {P}_{3}^{i,j}(x,t)& = {} P_{L,i}^{(\theta _{1},\vartheta _{1},{\alpha (x,t)})}(x)\, P_{T,j}^{(\theta _{2},\vartheta _{2})}(t). \end{aligned}$$

Now, adopting (18)–(21), enable one to write (15) in the form:

22 $$\begin{aligned} \begin{aligned} \sum \limits _{i,j = 0}^N\hat{u}_{i,j}\,\mathcal {P}_{2}^{i,j}(x,t)=B(x,t)\,\sum \limits _{i,j = 0}^N\hat{u}_{i,j}\,\mathcal {P}_{3}^{i,j}(x,t)+f\left( \sum \limits _{i,j=0}^N \hat{u}_{i,j}\,\mathcal {P}_{0}^{i,j}(x,t),x,t\right) , \end{aligned} \end{aligned}$$

while the numerical treatments of initial and boundary conditions are

23 $$\begin{aligned} \begin{aligned} u(x,0)=\sum \limits _{i,j = 0}^N\hat{u}_{i,j}\,\mathcal {P}_{0}^{i,j}(x,0)=g_{0}(x),\\ D_{t}u(x,0)=\sum \limits _{i,j = 0}^N\hat{u}_{i,j}\,\mathcal {P}_{1}^{i,j}(x,0)=g_{1}(x),\\ u(0,t)=\sum \limits _{i,j = 0}^N\hat{u}_{i,j}\,\mathcal {P}_{0}^{i,j}(0,t)=g_{2}(t),\\ u(L,t)=\sum \limits _{i,j= 0}^N\hat{u}_{i,j}\,\mathcal {P}_{0}^{i,j}(L,t)=g_{3}(t). \end{aligned} \end{aligned}$$

In the proposed shifted Jacobi collocation method, the residual of (15) is set to be zero at $(N-1)^2$ of collocation points. Moreover, the initial-boundary conditions in (23) will be collocated at collocation points. Firstly, we have $(N-1)^2$ algebraic equations for $(N+1)^2$ unknowns of $\hat{u}_{i,j}$

24 $$\begin{aligned} \begin{aligned} \sum \limits _{i,j = 0}^N\hat{u}_{i,j}\,F_{r,\tau }^{i,j}&=f\left( \sum \limits _{i,j=0}^N \hat{u}_{i,j}\,\mathcal {P}_{0}^{i,j}(x_{G,L,r}^{(\theta ,\vartheta )},t_{R,T,\tau }^{(\theta ,\vartheta )}),x_{G,L,r}^{(\theta ,\vartheta )},t_{R,T,\tau }^{(\theta ,\vartheta )}\right) ,\\ r&=1,\ldots ,N-1;\,\tau =1,\ldots ,N-1, \end{aligned} \end{aligned}$$

where

25 $$\begin{aligned} \begin{aligned} F_{r,\tau }^{i,j} &=\mathcal {P}_{2}^{i,j}\left( x_{G,L,r}^{(\theta ,\vartheta )},t_{R,T,\tau }^{(\theta ,\vartheta )}\right) -B\left( x_{G,L,r}^{(\theta ,\vartheta )},t_{R,T,\tau }^{(\theta ,\vartheta )}\right) \,\mathcal {P}_{3}^{i,j}\left( x_{G,L,r}^{(\theta ,\vartheta )},t_{R,T,\tau }^{(\theta ,\vartheta )}\right) \end{aligned} \end{aligned}$$

and also we have $2(N-1)$ algebraic equations which will be obtained due to the initial conditions

26 $$\begin{aligned} \begin{aligned} \sum \limits _{i,j, = 0}^N\hat{u}_{i,j,}\,\mathcal {P}_{0}^{i,j}\left( x_{G,L,r}^{(\theta ,\vartheta )},0 \right)&=g_{0}\left( x_{G,L,r}^{(\theta ,\vartheta )}\right) ,\quad r=1,\ldots ,N-1,\\ \sum \limits _{i,j, = 0}^N\hat{u}_{i,j,}\,\mathcal {P}_{1}^{i,j}\left( x_{G,L,r}^{(\theta ,\vartheta )},0 \right)&=g_{1}\left( x_{G,L,r}^{(\theta ,\vartheta )}\right) ,\quad r=1,\ldots ,N-1. \end{aligned} \end{aligned}$$

Furthermore, using the boundary conditions, we have $2(N+1)$ algebraic equations

27 $$\begin{aligned} \begin{aligned} \sum \limits _{i,j= 0}^N\hat{u}_{i,j}\,\mathcal {P}_{0}^{i,j}\left( 0,t_{R,T,\tau }^{(\theta ,\vartheta )}\right)&=g_{2}\left( t_{R,T,\tau }^{(\theta ,\vartheta )}\right) ,\quad \tau =0,\ldots ,N,\\ \sum \limits _{i,j = 0}^N\hat{u}_{i,j}\,\mathcal {P}_{0}^{i,j}\left( L,t_{R,T,\tau }^{(\theta ,\vartheta )}\right)&=g_{3}\left( t_{R,T,\tau }^{(\theta ,\vartheta )}\right) ,\quad \tau =0,\ldots ,N. \end{aligned} \end{aligned}$$

Combining Eqs. (24), (26) and (27), we obtain

28 $$\begin{aligned} {\left\{ \begin{array}{ll} \sum \nolimits _{i,j = 0}^N\hat{u}_{i,j}\,F_{r,\tau }^{i,j}=f\left( \sum \nolimits _{i,j=0}^N \hat{u}_{i,j}\,\mathcal {P}_{0}^{i,j}\left( x_{G,L,r}^{(\theta ,\vartheta )},t_{R,T,\tau }^{(\theta ,\vartheta )}\right) ,x_{G,L,r}^{(\theta ,\vartheta )},t_{R,T,\tau }^{(\theta ,\vartheta )}\right) ,\ & \quad r,\tau =1,\ldots ,N-1,\\ \sum \nolimits _{i,j, = 0}^N\hat{u}_{i,j,}\,\mathcal {P}_{0}^{i,j}\left( x_{G,L,r}^{(\theta ,\vartheta )},0 \right) =g_{0}\left( x_{G,L,r}^{(\theta ,\vartheta )}\right) ,\quad r=1,\ldots ,N-1,\\ \sum \nolimits _{i,j, = 0}^N\hat{u}_{i,j,}\,\mathcal {P}_{1}^{i,j}\left( x_{G,L,r}^{(\theta ,\vartheta )},0\right) =g_{1}\left( x_{G,L,r}^{(\theta ,\vartheta )}\right) ,\quad r=1,\ldots ,N-1,\\ \sum \nolimits _{i,j= 0}^N\hat{u}_{i,j}\,\mathcal {P}_{0}^{i,j}\left( 0,t_{R,T,\tau }^{(\theta ,\vartheta )}\right) =g_{2}\left( t_{R,T,\tau }^{(\theta ,\vartheta )}\right) ,\quad \tau =0,\ldots ,N,\\ \sum \nolimits _{i,j = 0}^N\hat{u}_{i,j}\,\mathcal {P}_{0}^{i,j}\left( L,t_{R,T,\tau }^{(\theta ,\vartheta )}\right) =g_{3}\left( t_{R,T,\tau }^{(\theta ,\vartheta )}\right) ,\quad \tau =0,\ldots ,N. \end{array}\right. } \end{aligned}$$

The previous system of nonlinear algebraic equations can be easily solved. After the coefficients $a_{i,j}$ are determined, it is straightforward to compute the approximate solution $u_{N,M}(x,t)$ at any value of (*x*, *t*) in the given domain from the following equation

29 $$\begin{aligned} u(x,t)=\sum \limits _{i,j=0}^N{\hat{u}_{i,j}\, P_{L,i}^{(\theta {_{1}},\vartheta _{1})}(x)\, P_{T,j}^{(\theta _{2},\vartheta _{2})}(t)}. \end{aligned}$$

## Numerical examples

This section reports two numerical examples to demonstrate the high accuracy and applicability of the proposed method. We also compare the results given from our scheme and those reported in the literature. The comparisons reveal that our method is very effective and convenient.

### *Example 1*

Consider the following variable order fractional wave equation which is given in Sweilam and Assiri (2015),

30 $$\begin{aligned} \begin{aligned} {}^{C}D_{t}^{\beta (x,t)}u(x,t)=-0.5\cos (\alpha (x,t)\pi /2){}^{C}D_{x}^{\alpha (x,t)}u(x,t)+f(u,x,t),\quad (x,t)\in [0,8]\times [0,1], \end{aligned} \end{aligned}$$

where $$\beta (x,t)=1.5+0.25\cos (x)\sin (2t),\,\,\alpha (x,t)=1.5+0.5 e^{-(xt)^{2}-1}$$ and

$$\begin{aligned} f(u,x,t)=\frac{2u}{t^{2}+1}-(t^{2}+1)\left( \,\frac{16 x^{2-\alpha (x,t)}}{\Gamma (3-\alpha (x,t))}+\frac{6 x^{3-\alpha (x,t)}}{\Gamma (4-\alpha (x,t))}\right) , \end{aligned}$$

with the initial-boundary conditions

31 $$\begin{aligned} \begin{aligned} u(x,0)=x^{2}(8-x),\quad u_{t}(x,0)=0,\quad u(0,t)=u(8,t)=0, \quad (x,t)\in [0,8]\times [0,1]. \end{aligned} \end{aligned}$$

The exact solution of this problem when $\alpha (x,t)=\beta (x,t)=2$ is given by

32 $$\begin{aligned} \begin{aligned} u(x,t)=x^{2}(8-x)(t^{2}+1),\quad (x,t)\in [0,8]\times [0,1]. \end{aligned} \end{aligned}$$

Sweilam and Assiri (2015) proposed the non-standard finite difference (NSFD) method to solve this problem with choices of $N=1000$ and $M=125$. In Table 1, we contrast our numerical results based on absolute errors obtained using the proposed algorithm for three choices of the shifted Jacobi parameters at $N=8$ with the corresponding results of NSFD method (Sweilam and Assiri 2015). In Table 2, we contrast our results based on maximum absolute errors (MAEs) obtained by the present method for three choices the shifted Jacobi parameters at $N=8$. From the results of this example, we observe that the approximate solution obtained by our method is more better than those obtained in Sweilam and Assiri (2015).

**Table 1:** The absolute errors of problem (30) for our method at $N=8$ and the NSFD method in Sweilam and Assiri (2015)

*x*	*t*	Our method			NSFD method (Sweilam and Assiri 2015)
		$\theta _{1}=\theta _{2}=0$	$\theta _{1}=\theta _{2}=\frac{1}{2}$	$\theta _{1}=1,\theta _{2}=0$	$N=1000$
		$\vartheta _{1}=\vartheta _{2}=0$	$\vartheta _{1}=\vartheta _{2}=\frac{1}{2}$	$\vartheta _{1}=0,\vartheta _{2}=1$	$M=125$
0	1	2.15204 × 10^−14^	3.55271 × 10^−14^	0	0
0.8		2.1985 × 10^−15^	9.9476 × 10^−14^	3.55271 × 10^−14^	2.70905 × 10^−3^
1.6		1.22734 × 10^−14^	1.84741 × 10^−13^	1.42109 × 10^−14^	2.47795 × 10^−3^
2.4		1.60228 × 10^−14^	5.68434 × 10^−14^	7.10543 × 10^−14^	2.12119 × 10^−3^
3.2		3.54371 × 10^−15^	1.42109 × 10^−14^	1.42109 × 10^−14^	1.62219 × 10^−3^
4		1.69769 × 10^−14^	2.84217 × 10^−14^	2.84217 × 10^−14^	9.1329 × 10^−4^
4.8		1.93105 × 10^−14^	8.52651 × 10^−14^	5.68434 × 10^−14^	9.988 × 10^−5^
5.6		5.34145 × 10^−14^	0	0	1.52948 × 10^−3^
6.4		2.16945 × 10^−14^	1.42109 × 10^−14^	2.13163 × 10^−14^	3.50169 × 10^−3^
7.2		5.49134 × 10^−14^	3.55271 × 10-15	0	6.15396 × 10^−3^
8		1.37362 × 10^−15^	1.42109 × 10^−14^	0	0

**Table 2:** The MAEs of problem (30) for our method at $N=8$ and the NSFD method in Sweilam and Assiri (2015)

*T*	Our method			NSFD method (Sweilam and Assiri 2015)
	$\theta _{1}=\theta _{2}=0$	$\theta _{1}=\theta _{2}=\frac{1}{2}$	$\theta _{1}=1,\theta _{2}=0$	$N=1000$
	$\vartheta _{1}=\vartheta _{2}=0$	$\vartheta _{1}=\vartheta _{2}=\frac{1}{2}$	$\vartheta _{1}=0,\vartheta _{2}=1$	$M=125$
1	$3.88506\times 10^{-14}$	$3.55271\times 10^{-14}$	$5.68434\times 10^{-14}$	$6.1539\times 10^{-3}$
4	$4.86067\times 10^{-13}$	$6.82121\times 10^{-13}$	$7.95808\times 10^{-13}$	$3.4818\times 10^{-3}$
8	$2.00448\times 10^{-11}$	$5.00222\times 10^{-12}$	$6.36646\times 10^{-12}$	$9.0641\times 10^{-5}$

**Fig. 1:**
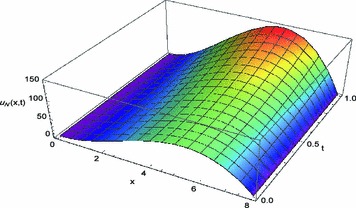
Space-graph of numerical solution of problem (1)

**Fig. 2:**
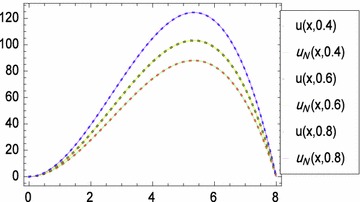
*x*-Direction curves of exact and numerical solutions of problem (1)

**Fig. 3:**
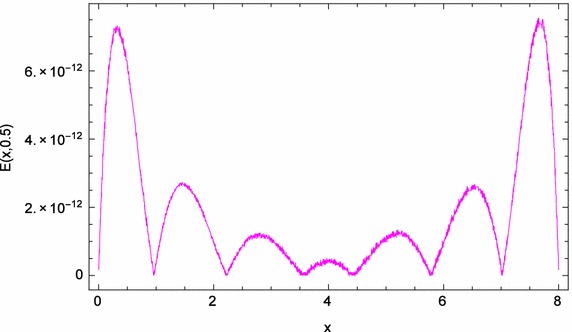
*x*-Direction curve of the absolute errors of problem (1)

**Fig. 4:**
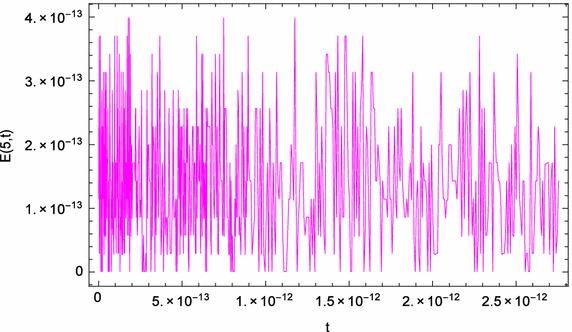
*t*-Direction curve of the absolute errors of problem (1)

Figure 1 displays the space-graph of the numerical solution of problem (1) with $N=8,$ and $\theta _{1}=\theta _{2}=\vartheta _{1}=\vartheta _{2}=0$. While, Fig. 2 compares graphically the curves of numerical and exact solutions of problem (1) for the different values of *t* at $N=8,$ and $\theta _{1}=\theta _{2}=\vartheta _{1}=\vartheta _{2}=\frac{1}{2}$. Moreover, we represent in Figs. 3 and 4 the absolute error curves obtained by the present method at $t=0.5$ and $x=5$ with $N=8,$ and $\theta _{1}=\theta _{2}=\vartheta _{1}=\vartheta _{2}=0$, respectively. This demonstrates that the proposed method leads to an accurate approximation and yields exponential convergence rates.

### *Example 2*

Consider the following problem

33 $$\begin{aligned} \begin{aligned} {}^{C}D_{t}^{\beta (x,t)}u(x,t)=t^2\,{}^{C}D_{x}^{\alpha (x,t)}u(x,t)+f(x,t),\quad (x,t)\in [0,1]\times [0,1], \end{aligned} \end{aligned}$$

where

$$\begin{aligned}\begin{aligned} \beta (x,t)=2-\sin ^{2}(t) \cos ^{2}(x),\quad \alpha (x,t)=1.8\, +0.5 e^{-(t x)^2-1}. \end{aligned} \end{aligned}$$

with the initial and boundary conditions

34 $$\begin{aligned} \begin{aligned} u(x,0)=0,\quad u_{t}(x,0)=0,\quad u(0,t)=t^3,\quad u(1,t)=t^3, \quad (x,t)\in [0,1]\times [0,1], \end{aligned} \end{aligned}$$

where *f*(*x*, *t*) is a given function such that the exact solution of this problem is

35 $$\begin{aligned} \begin{aligned} u(x,t)=t^3 \cos (2 \pi x),\quad (x,t)\in [0,1]\times [0,1]. \end{aligned} \end{aligned}$$

In Table 3, we list the results based on the MAEs obtained by the proposed method (with various choices of $N, \theta _{1},\, \theta _{2},\,\vartheta _{1},$ and $\vartheta _{2}$). From this table, we see that we can achieve an excellent approximation for the exact solution by using proposed method for a limited number of the collocation nodes. Also this demonstrates that the proposed method provides an accurate approximation and yields exponential convergence rates.

**Table 3:** The MAEs of problem 2

*N*	Our method with several choices of $N,\,M$		
	(0, 0, 0, 0)	$\left(\frac{1}{2},\frac{1}{2},\frac{1}{2},\frac{1}{2}\right)$	$\left(-\frac{1}{2},-\frac{1}{2},\frac{1}{2},\frac{1}{2}\right)$
4	$7.3277\times 10^{-2}$	$4.46797\times 10^{-2}$	0.107228
8	$1.024\times 10^{-4}$	$1.09006\times 10^{-4}$	$1.14665\times 10^{-4}$
12	$1.55737\times 10^{-8}$	$2.31041\times 10^{-8}$	$3.18938\times 10^{-8}$
16	$1.75149\times 10^{-12}$	$3.27383\times 10^{-12}$	$2.14628\times 10^{-12}$
20	$3.70814\times 10^{-14}$	$5.32907\times 10^{-14}$	$6.51701\times 10^{-14}$

**Fig. 5:**
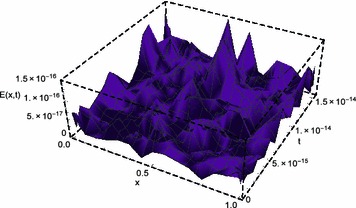
space graph of the absolute errors of problem (2)

**Fig. 6:**
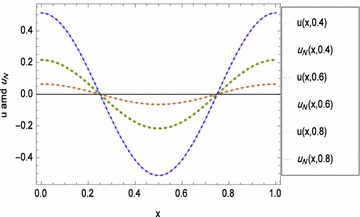
*x*-Direction curves of exact and numerical solutions of problem (2)

**Fig. 7:**
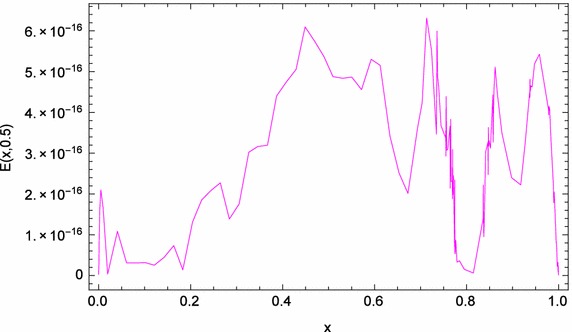
*x*-Direction curve of the absolute errors of problem (2)

**Fig. 8:**
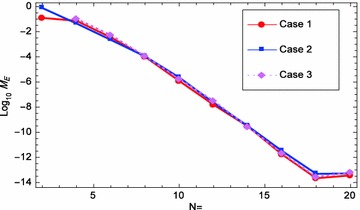
$M_E$ convergence of problem (2)

Figure 5 shows the space graph of the absolute errors with $N=20,$ and $\theta _{1}=\theta _{2}=-\frac{1}{2},\,\vartheta _{1}=\vartheta _{2}=\frac{1}{2}$. While, Fig. 6 compares graphically the curves of numerical and exact solutions of problem (2) for the different values of *t* at $N=20, \theta _{1}=\theta _{2}=-\frac{1}{2},\,\vartheta _{1}=\vartheta _{2}=\frac{1}{2}$. Meanwhile, we plot in Fig. 7 the absolute error curve obtained by the present method at $t=0.5$ with $N=20,$ and $\theta _{1}=\theta _{2}=\vartheta _{1}=\vartheta _{2}=0$. Moreover, we present in Fig. 8 the logarithmic graphs of MAEs (i.e., $log_{10} M_E$) obtained by the present method with different values of $(N=2,4, 6, \cdots , 20)$ at three cases of $\theta _{1},\,\theta _{2},\,\vartheta _{1},$ and $\vartheta _{2}$

1. Case 1, $$\theta _{1}=\theta _{2}=\vartheta _{1}=\vartheta _{2}=0$$.
2. Case 2, $$\theta _{1}=\theta _{2}=\vartheta _{1}=\vartheta _{2}=\frac{1}{2}$$.
3. Case 3, $$\theta _{1}=\theta _{2}=-\frac{1}{2}, \,\vartheta _{1}=\vartheta _{2}=\frac{1}{2}$$.

All the above numerical simulations demonstrate the high accuracy and applicability of our technique.

## Conclusions

We presented a collocation method to achieve an accurate numerical solution for variable-order fractional wave problem subject to initial-boundary conditions. One of the most advantages of the present technique is that a fully spectral method was implemented for the time and space variables by using SJ–GR-C and SJ–G-C approximations respectively. The problem with its conditions was then reduced to an algebraic system. The greatest feature of the present scheme is, adding few terms of the SJ–G and SJ–GR collocation points, a full agreement between the approximate and exact solutions was achieved. Through the numerical examples and specially the comparison between the obtained approximate solution and those obtained by other approximations, we demonstrate the validity and high accuracy of the present method.
